# Changes in metabolites in raw and wine processed Corni Fructus combination metabolomics with network analysis focusing on potential hypoglycemic effects

**DOI:** 10.3389/fphar.2023.1173747

**Published:** 2023-08-07

**Authors:** Siqian Zhou, Jian Liu, Leihong Tan, Yikun Wang, Jing Li, Yajing Wang, Changsong Ding, Hongping Long

**Affiliations:** ^1^ Center for Medical Research and Innovation, The First Hospital of Hunan University of Chinese Medicine, Changsha, China; ^2^ Hunan University of Chinese Medicine, Changsha, China; ^3^ Department of Pharmacy, The Second Hospital of Hunan University of Chinese Medicine, Changsha, China; ^4^ Department of Pharmacy, The Second Xiangya Hospital, Central South University, Changsha, China; ^5^ Department of Pharmacy, Xiangya Hospital, Central South University, Changsha, China

**Keywords:** Corni Fructus, metabolomics, network analysis, hypoglycemic, wine-processed markers

## Abstract

**Introduction:** Corni Fructus (CF) is a Chinese herbal medicine used for medicinal and dietary purposes. It is available commercially in two main forms: raw CF (unprocessed CF) and wine-processed CF. Clinical observations have indicated that wine-processed CF exhibits superior hypoglycemic activity compared to its raw counterpart. However, the mechanisms responsible for this improvement are not well understood.

**Methods:** To address this gap in knowledge, we conducted metabolomics analysis using ultra-performance liquid chromatography-quadrupole/time-of-flight mass spectrometry (UPLC-QTOF-MS) to compare the chemical composition of raw CF and wine-processed CF. Subsequently, network analysis, along with immunofluorescence assays, was employed to elucidate the potential targets and mechanisms underlying the hypoglycemic effects of metabolites in CF.

**Results:** Our results revealed significant compositional differences between raw CF and wine-processed CF, identifying 34 potential markers for distinguishing between the two forms of CF. Notably, wine processing led to a marked decrease in iridoid glycosides and flavonoid glycosides, which are abundant in raw CF. Network analysis predictions provided clues that eight compounds might serve as hypoglycemic metabolites of CF, and glucokinase (GCK) and adenylate cyclase (ADCYs) were speculated as possible key targets responsible for the hypoglycemic effects of CF. Immunofluorescence assays confirmed that oleanolic acid and ursolic acid, two bioactive compounds present in CF, significantly upregulated the expression of GCK and ADCYs in the HepG2 cell model.

**Discussion:** These findings support the notion that CF exerted hypoglycemic activity via multiple components and targets, shedding light on the impact of processing methods on the chemical composition and hypoglycemic activity of Chinese herbal medicine.

## 1 Introduction

Corni Fructus (CF; *shanzhuyu* in Chinese), derived from the dried mature fruits of *Cornus officinalis* Sieb. et Zucc., which had been widely used in traditional Chinese medicine (TCM) in Asia to treat multiple diseases (Cui et al., 2021). As a TCM, CF has been extensively utilized in China to treat diabetes by nourishing the liver and kidney, addressing kidney deficiency, regulating hypertension and other related diseases (Gao et al., 2021). Moreover, CF is an important medicinal component in many classic TCM prescriptions, such as Liuwei Dihuang pill and Zuogui pill (Zhou et al., 2020). Modern pharmacological studies have indicated that CF exhibits a broad spectrum of pharmacological activities, including hypoglycemic and hypolipidemic activity, liver and kidney protection, and other activities (Huang et al., 2018). Phytochemical research revealed that active components of CF majorly include iridoids, flavonoids, and triterpenes, which are employed for antioxidative, antidiabetic, and antineoplastic activities (Ma et al., 2014; Dong et al., 2018).

Processing is an essential step in TCM preparation, which can alter the properties of medicinal substances, reduce TCM toxicity, and enhance TCM efficacy (Zhao et al., 2010). In addition, as two commercial products, there are differences in pharmacological activity between raw CF and wine-processed CF. Long-term clinical practice has shown that compared to raw CF, wine-processed CF has stronger effects on nourishing the liver and kidney, and exhibits superior hypoglycemic activity (Zhang et al., 2016; Bi et al., 2019). However, the bioactive chemical changes occurring during the wine processing of CF remain unclear.

Currently, several studies have been carried out to analyze the changes in components between raw CF and wine-processed CF (Cao et al., 2020; Han et al., 2022). Previous studies revealed that several iridoids showed significant differences in raw CF and wine-processed CF by HPLC-MS (Wang et al., 2018). However, these previous studies were only low throughput analyses, fail to systematically illustrate the chemical alteration involved in the wine processing of CF, and it is difficult to screen wine-processing associated markers due to the chemical complexity of CF. LC-MS based metabolomics is a valuable approach for high-throughput detection and analysis of secondary metabolites and active ingredients in medicinal plants (Xie et al., 2022). Moreover, with the aid of multivariate statistical analysis, metabolomics could screen meaningful markers for reflecting chemical varieties caused by TCM processing (Xia et al., 2020; Gao et al., 2022).

**FIGURE 1:**
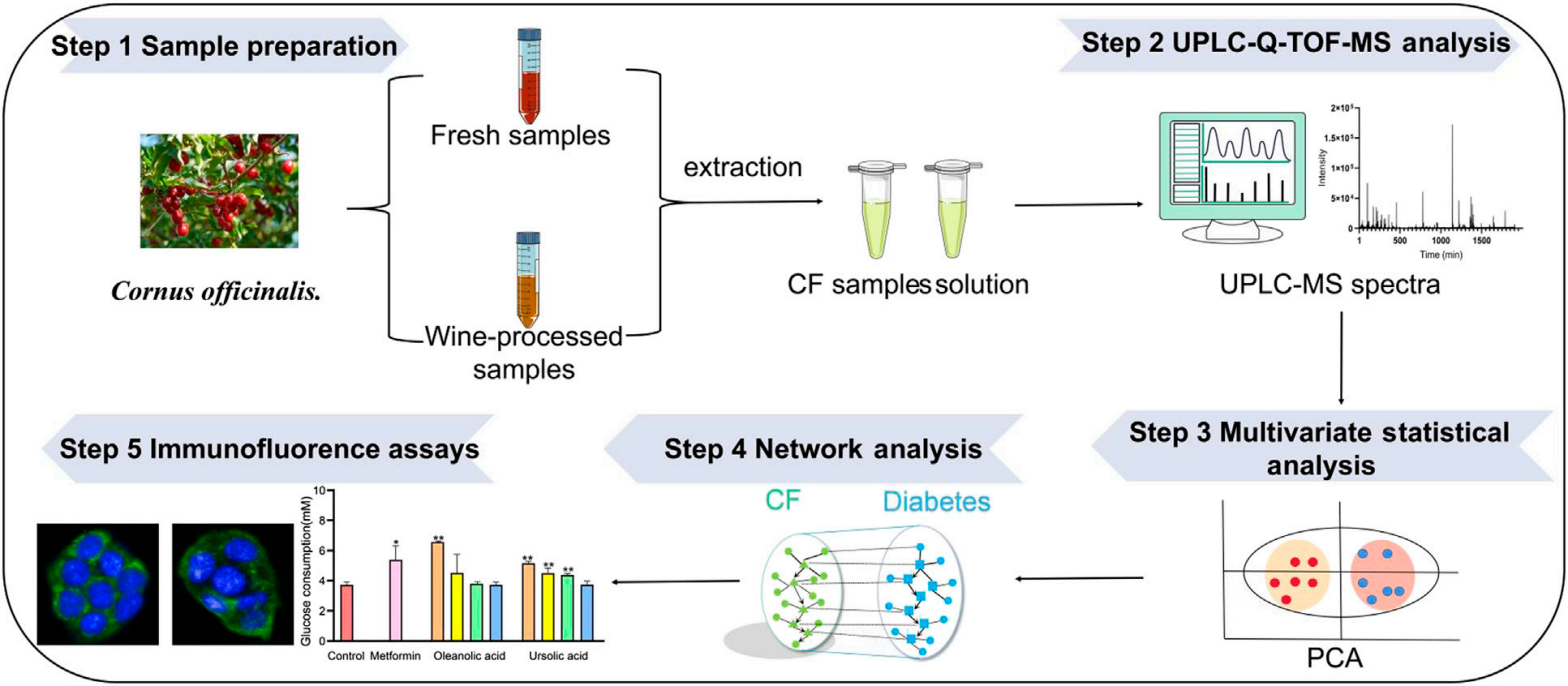
The strategy to discover potential hypoglycemic metabolites based on metabolomics and network analysis.

In the present study, an integrated strategy was established to unveil the changes in hypoglycemice metabolites in raw CF and wine-processed CF. Firstly, ultra-high performance liquid chromatography-quadrupole time-of-flight mass spectrometry (UPLC/Q-TOF-MS) based metabolomics was performed to compare the plant metabolic profiling and metabolites changes of 20 batches CF samples, and differential metabolites responsible for distinguishing raw CF and wine-processed CF was screened. Then, the active ingredients of CF and their hypoglycemic potential targets were predicted by network analysis. Finally, immunofluorescence assays and quantitative analysis were applied to further verify the hypoglycemic mechanism of CF in the HepG2 cells model (Figure 1).

## 2 Materials and methods

### 2.1 Chemicals and reagents

Geniposide (Lot: 110749-201919), loganin (Lot: 110640-201707), morroniside (Lot:111998-201703), rutin (Lot: 100080-201811), quercetin (Lot: 100081-201610), kaempferol (Lot: 110861-202013), and caffeic acid (Lot: 110885-201703) were all bought from China National Institutes for Food and Drug Control (Beijing, China). Gallic acid (Lot: 190715-008), 5-hydroxymethylfurfural (Lot: 191015-037) and cornuside (Lot: 190917-066) were purchased from Beijing Ya Xi’er Technology Co., Ltd. (Beijing, China). Astragalin (Lot: 19092602) was purchased from Chengdu Herbpurify (Chengdu, China). LC-MS-grade acetonitrile and methanol were supplied by Merck (Darmstadt, Germany), and LC-MS-grade formic acid was purchased from Acslabchem (ACS, United States). Ultrapure water was supplied by Shenzhen Watsons Distilled Water Co., Ltd.

CF samples were purchased from five herbal pieces factories in China and identified by Professor Zhiguo Zhang from The First Hospital of Hunan University of Chinese Medicine. The specific information is shown in Supplementary Table S1.

### 2.2 Sample preparation

According to the wine-processing methods of CF recorded in the Chinese Pharmacopoeia (National Commission of Chinese Pharmacopoeia, 2020 version), the raw CF was mixed with wine, saturated, and the temperature set up at 115°C, then steamed with high-pressure wine for 1 h, dried for 4 h at 60°C. Finally, it is removed for cooling, wine-processed CF was prepared.

Each batch of CF sample was weighed 100 g, extracted with 8 volumes of water and refluxed twice for 1 h each at 100°C. And the CF water extracts was merged, concentrated in vacuum, and subsequently lyophilized to prepare a CF extract powder. The 3 g powder was weighed precisely, and then was dissolved in a 30 mL 50% methanol. The solution was sonicated for 30 min, centrifugated, filtered and obtained the CF sample solution which was used for LC/MS analysis.

Eleven reference standards, including gallic acid, geniposide, loganin, morroniside, rutin, quercetin, kaempferol, 5-hydroxymethylfurfural, caffeic acid, cornuside and astragalin, were accurately weighed 10 mg, added in a 25 mL volumetric bottle, and dissolved in methanol yielding a standard solution at 0.4 mg/mL.

### 2.3 UPLC-Q-TOF-MS conditions

LC-MS/MS (1290UPLC-6540-QTOF, Agilent, United States) was applied to qualitatively analyze the metabolites in CF, a high-efficiency C18 column (3.0 × 100 mm, 1.8 μm, Agilent) was used to separate metabolites, the flow rate was set at 0.4 mL/min, and the separation was subjected to gradient elution mode. The mobile phase consisted of water (included 0.1% formic acid, A) and acetonitrile (B), the elution conditions are described in the Supplementary Materials. ESI positive and negative ion mode were adopted in the mass spectrum, LC-MS analysis methods were used according to our previously published article (Wang et al., 2020b). Molecule Feature Extractor of Masshunter Qualitative Analysis (Agilent, United States) was applied to analyze the primary and secondary mass spectrometry data. The identification of metabolites in CF were conducted through comparison with standards and MS/MS fragmentation and GNPS platform. UPLC-DAD was further performed to quantitatively analyze metabolites.

### 2.4 Multivariate statistical analysis screened

The LC-MS raw data of raw CF and wine-processed CF samples were imported to MassHunter Profinder (Agilent, United States), and converted mass spectrometry data into the matrix format of metabolite peak area, and peak alignment and matching was performed. Moreover, multivariate statistical analysis (Simca-p14.0 software, Umetrics AB, Sweden) was adopted to analyze to all the resultant data matrix. Hierarchical cluster analysis (HCA) and principal component analysis (PCA) as unsupervised pattern recognition methods were used to cluster analysis to distinguish metabolic phenotypes between raw CF and wine-processed CF. To screen markers associated with wine processing more effectively, OPLS-DA was used to observe the main characteristic ingredient for the data variance. The variable importance parameter (VIP > 1) value of the validated OPLS-DA model and *p* < 0.05 in the Student’s test were taken as candidate distinguishing markers. Finally, the structures of metabolites were determined by analyzing the elemental compositions and MS/MS fragmentation, and compared the retention time of samples with authentic standards. GNPS (https://gnps.ucsd.edu/ProteoSAFe/static/gnps-splash.jsp), PubChem (https://pubchem.ncbi.nlm.nih.gov/) and references were used for the annotation of distinguishing metabolites.

### 2.5 Network analysis

The targets of wine-processed CF metabolites were predicted from the TCMSP database and the Swiss Target Prediction platform, the species was set *Homo sapiens* (Dai et al., 2016; Gan et al., 2021). Then, all targets of CF metabolites were merged, and standardized into official gene names via the UniProt database (Pundir et al., 2016). The disease-related genes were screened out by DisGeNET (Piñero et al., 2020), GeneCards (Fishilevich et al., 2016), and OMIM (Hamosh et al., 2021). Using the Venn diagram to obtain the intersection targets between metabolites targets and diabetes targets. The intersection targets were uploaded to the STRING database (Szklarczyk et al., 2023) to construct the protein-protein interaction (PPI) network. Cytoscape software (version 3.9.2) was applied to analyze the PPI network, the hub hypoglycemic targets of CF, according to the topological parameters of “degree”. The KEGG pathway and Gene Ontology (GO) enrichments of potential targets were analyzed by the DAVID database (Huang et al., 2007), “*Homo sapiens*” and *p* < 0.05 was selected as the standard of the KEGG pathway.

### 2.6 Cell culture and treatment

HepG2 cells (Procell Life Science & Technology Co., Ltd., China, CL-0103) were cultured at 37°C in 5% CO_2_, the medium contained 10% fetal bovine serum (FBS) supplemented with 100 U/mL penicillin and 100 g/mL streptomycin (HyClone, United States) in high glucose Dulbecco’s Modified Eagle Medium (DMEM) (Gibco, United States), HepG2 cells in logarithmic phase were seeded into 96-well plates for 24 h.

### 2.7 Effect of CF metabolites on the viability of HepG2 cells

Cell viability was examined using the CCK-8 kit (Bioss, China). Briefly, HepG2 cells in the logarithmic phase were cultured into 96-well plates for 24 h. Then, the medium was replaced with DMEM culture medium without FBS containing metabolites groups at different molar concentrations (10 *μ*M, 1 *μ*M, 0.1 *μ*M, 0.01 *μ*M) for 24 h. Ten tested groups were divided into control group, 0.5% DMSO (vehicle), kaempferol, oleanolic acid, loganin, quercetin, ursolic acid, morroniside and active metabolites of CF (six standard solutions were mixed in equal proportions). Next, 10 *µ*L of CCK-8 was added to each well and cultured at 37°C for 1 h. The optical density was measured at 450 nm using a microplate reader (PerkinElmer, Enspire).

### 2.8 Glucose consumption for normal HepG2 cells

HepG2 cells in logarithmic phase were seeded into 96-well plates for 24 h, DMEM culture medium without FBS was replaced and cultured for 12 h, then the medium was discarded, and the culture medium of drug groups was added for 24 h. Furthermore, the content of glucose in the supernatant of the culture medium was tested with a glucose detection kit (Robio, China), glucose consumption = glucose content of blank group-glucose content of drug group.

### 2.9 Immunofluorescence assay and quantitative analysis

Treated HepG2 cells were incubated for 24 h, washed with PBS three times, fixed with fixative for 45 min, and treated with 0.25% triton-100 for 15 min. Then, blocked with 5% bovine serum albumin for 20 min, incubated with anti-GCK (1:100) and anti-ADCYs (ADCY2, ADCY3, ADCY8 and ADCY9) (1:100) in the dark overnight in a humidified container at 4°C. Following incubation with sheep anti-rabbit IgG (H + L) secondary antibody, cells were subsequently stained with DAPI. The protein expression level and images were observed in laser scanning confocal microscopy.

### 2.10 Statistical analysis

Statistical analysis was performed by SPSS 25.0, and mean ± standard deviation was used to express the data. The comparison between groups was conducted by one-way analysis of variance (ANOVA). *p* < 0.05 indicated that the difference was statistically significant.

## 3 Results

### 3.1 Chemical profiling of CF

**FIGURE 2:**
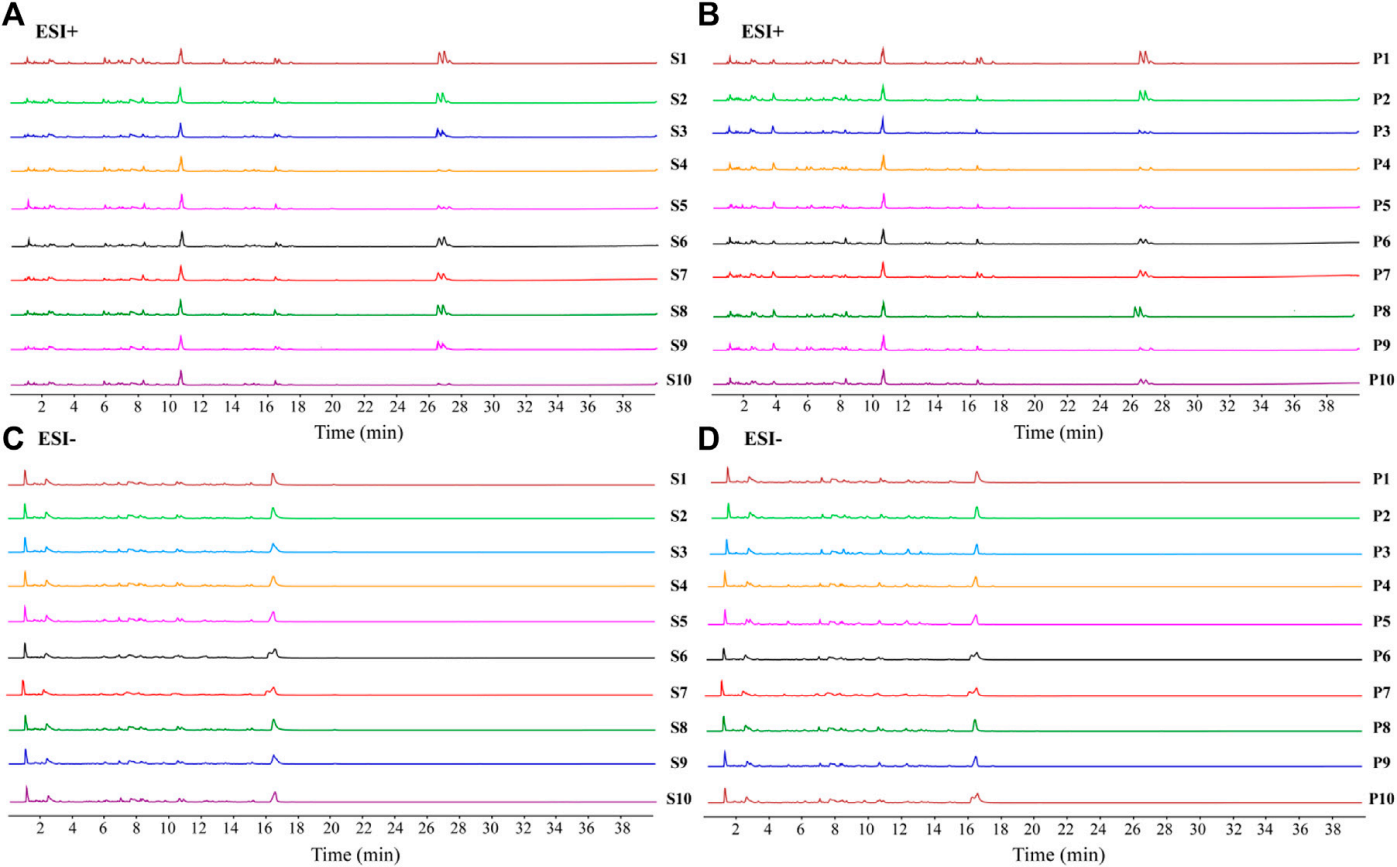
UPLC fingerprints of different batches of CF extracts. **(A)** raw CF in positive ion mode; **(B)** wine-processed CF in positive ion mode; **(C)** raw CF in negative ion mode; **(D)** wine-processed CF in negative ion mode.

The fingerprint of raw CF and wine-processed CF was established, as depicted in Figures 2A–D. A total of 20 batches of CF samples were analyzed using the software Chinese Medicine Fingerprint Similarity Evaluation (2012A version) to calculate the similarity values between raw CF and wine-processed products, The similarity index ranged from 0.745 to 0.984, indicating substantial differences between raw CF and wine-processed CF. By comparing with reference standards, a total of 11 metabolites were identified. This suggests that the chemical composition of CF varies among different batches. The results are presented in Supplementary Table S2.

### 3.2 Multivariate statistical analysis screened the distinguishing markers between raw CF and wine-processed CF samples

**FIGURE 3:**
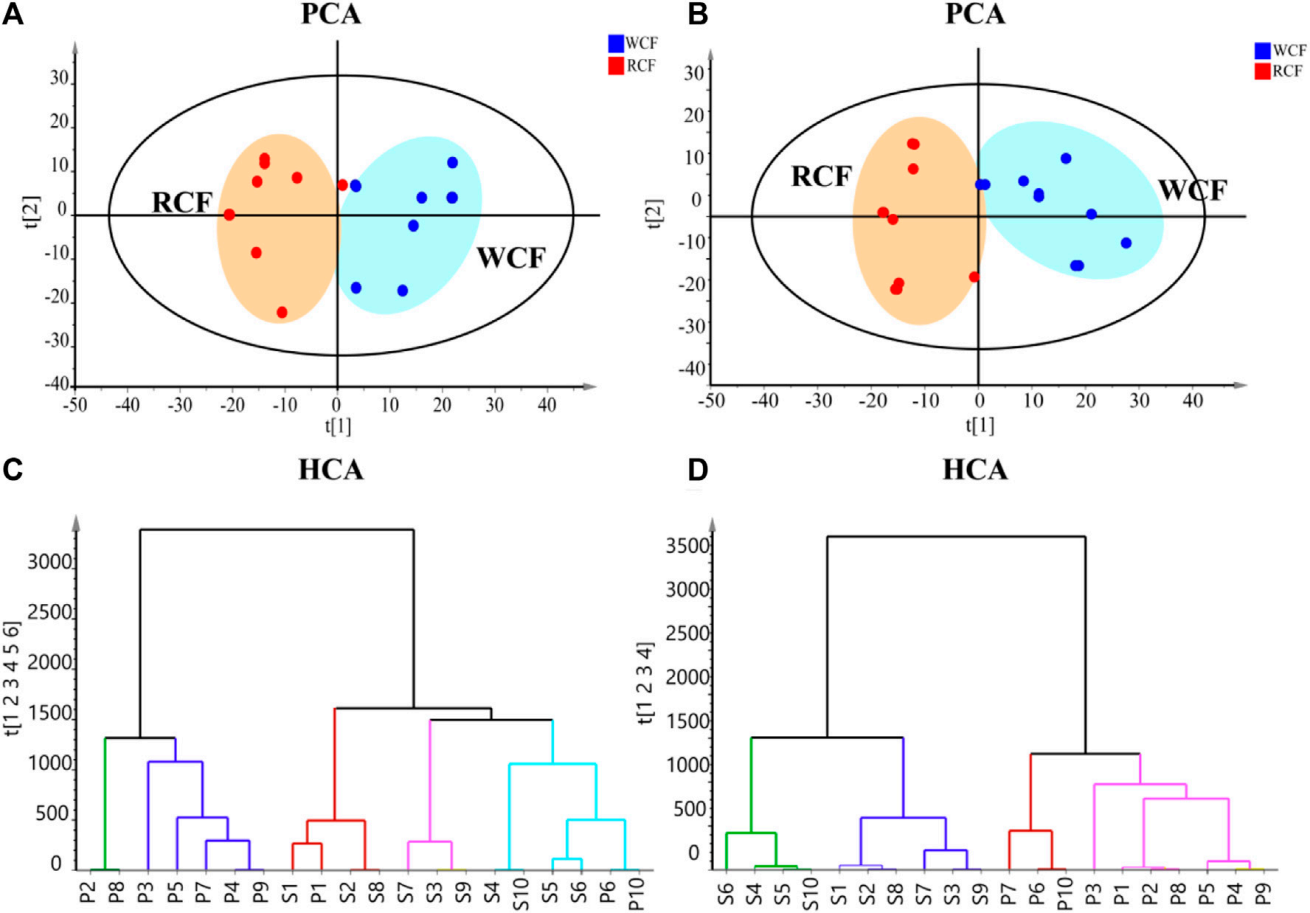
Metabolic phenotype differentiation between raw and wine-processed CF. **(A)** Positive ion mode of PCA score plot (R^2^X = 0.745, Q^2^ = 0.275). **(B)** Negative ion mode of PCA score plot (R^2^X = 0.689, Q^2^ = 0.410). **(C)** HCA model of raw and wine processed CF in positive ion mode. **(D)** HCA model of raw and wine-processed CF in negative ion mode.

To visualize metabolic differences between raw CF and wine-processed CF, we conducted unsupervised principal component analysis (PCA) in both positive and negative ion modes. As shown in Figures 3A, B, the PCA score plot clearly segregated the raw CF and wine-processed CF samples into distinct clusters in both ion modes, indicating that the processing methods induce chemical transformations in CF. The PCA model parameters were R^2^X = 0.745 and Q^2^ = 0.275 in the positive ion mode, and R^2^X = 0.689 and Q^2^ = 0.410 in the negative ion mode, suggesting the adequacy of the constructed model. Hierarchical cluster analysis (HCA) was used to evaluate the similarity of the differential markers (Figures 3C, D). The results demonstrated that all experimental samples were effectively separated into two main clusters in negative ion mode, whereas in the positive ion mode, S5 and P10 exhibited complete clustering with no distinguishable differences. Both HCA and PCA model suggested that processing altered chemical composition of CF.

To screen distinctive markers between raw CF and wine-processed CF, we employed OPLS-DA for further analysis. The OPLS-DA score plots demonstrated a clear separation of raw CF and wine-processed CF samples in both positive ion mode (R^2^Y = 0.995, Q^2^ = 0.938, Supplementary Figure S5) and negative ion mode (R^2^Y = 0.993, Q^2^ = 0.935, Supplementary Figure S5). Moreover, the metabolites accountable for the differences in CF processing were analyzed using the variable projection (VIP) value in the OPLS-DA model. Metabolites with VIP >1 in the model, along with a significant difference in Student's *t*-test (*p* < 0.05), were selected as criteria to identify differential markers. Finally, the molecular mass, MS/MS data, and standards were used to determine the chemical structures of the identified metabolites, leading to 19 markers in the positive ion mode and 20 markers in the negative ion mode (Table 1).

**TABLE 1:** Identification of metabolites in raw CF and wine-processed CF samples by UPLC-QTOF-MS/MS.

Peak ID	Rt (min)	Identification	Molecular	Molecular	Theoretical	Error	MS/MS fragment	VIP	*P*	Trend
			Formula	Ions	Exact mass	(ppm)				
1	1.15	Daphnin	C_15_H_16_O_9_	[M + Na]^+^	363.0697	−3.60	+: 346.9664, 333.7759,282.2018	2.0453	3.98E-08	↓
2	1.45	3-Phenylpropanal	C_9_H_8_O	[M + NH4]^+^	150.0915	−1.11	+: 122.0542, 109.0287, 97.0282	2.0210	2.56E-07	↓
3	1.67	Naringenin	C_15_H_12_O_5_	[M − H]^−^	271.0604	2.09	−: 179.8501,151.0150,119.0602	1.3224	0.019737	↓
4	2.67	Gallic acid	C_7_H_6_O_5_	[M + H]^+^	171.0282	3.27	+: 139.0424,111.0438,93.0341	1.4870	0.048389	↑
5	3.86	Theogallin	C_14_H_16_O_10_	[M + H]^+^	345.0806	1.09	+: 257.0238,153.0186,125.0321	1.9985	1.04E-06	↓
6	4.22	2,3-di-O-galloyl-D-glucose	C_20_H_20_O_14_	[M − H]^−^	483.0785	−0.81	−: 331.0788,271.0599,169.0259	1.4300	0.000065	↓
7	5.91	Loganic acid	C_16_H_24_O_10_	[[M − H]^−^	375.1295	−2.07	−: 213.0891,169.0986,113.0354	1.3784	0.001739	↓
8	6.57	Neoisoastilbin	C_21_H_22_O_11_	[M + CH_3_COO]^-^	509.1316	−3.17	−: 329.0771,271.0578,205.0262	1.3913	0.013253	↓
9	6.72	Sarracenin	C_11_H_14_O_5_	[M + H]^+^	227.0917	−2.28	+: 177.0551,155.0343,139.0394	1.6624	0.000040	↓
10	6.80	Morroniside	C_17_H_26_O_11_	[M − H]^−^	405.1409	−1.56	−: 243.1020,155.0463,101.0352	1.2161	0.009525	↑
11	7.54	Geniposide	C_17_H_24_O_10_	[M + H]^+^	389.1445	0.12	+: 227.1225,161.0623,111.0394	1.4721	0.011809	↑
12	8.53	3,4-di-O-galloylquinic acid	C_21_H_20_O_14_	[M + H]^+^	497.0945	−3.4	+: 416.7445,291.0499,153.0178	1.2052	0.031532	↑
13	9.85	Cornuside Ⅲ	C_21_H_30_O_14_	[M − H]^−^	505.1566	−0.60	−: 389.1476,227.1046,101.0349	1.2801	0.017282	↓
14	10.69	Naringenin-7-O-glucoside	C_21_H_22_O_10_	[M + COOH]^−^	479.1202	−1.30	−: 317.0740,205.0262,151.0180	1.4963	0.000680	↓
15	10.75	Tartaric acid	C_4_H_6_O_6_	[M − H]^−^	149.0092	0.31	−: 105.0312,87.0190,73.0020	1.6374	4.16E-06	↑
16	10.88	Loganin	C_17_H_26_O_10_	[M + Na]^+^	413.1420	−0.40	+: 229.1070,179.0695,151.0744	1.5386	0.000064	↓
				[M + COOH]−	435.1518		−: 227.1046,127.0511,101.0348			
17	11.31	1,2,3-tri-O-galloyl-β-D-glucose	C_27_H_24_O_18_	[[M − H]^−^	635.0896	−0.74	−: 465.0778,313.0686,169.0259	1.0093	0.001452	↑
18	12.09	Caffeic acid	C_9_H_8_O_4_	[M + H]^+^	181.0493	0.59	+: 168.0809,153.0556,139.0375	1.9099	0.003414	↑
19	13.08	2-hydroxycinnamic acid	C_9_H_8_O_3_	[M − H]^−^	163.0395	3.56	−: 145.0352,119.0608,93.0450	1.4905	0.002891	↑
20	13.61	Cornuside	C_24_H_30_O_14_	[M − H]^−^	541.1564	0.05	−: 379.1038,183.0296,169.0150	1.0203	0.044270	↓
21	13.73	Quercetin-3-O-arabino-glucoside	C_26_H_28_O_16_	[M − H]^−^	595.1307	−0.79	−: 417.2429,300.0262,209.0527	1.6979	6.54E-09	↑
22	13.78	Quercetin-3-D-xyloside	C_20_H_18_O_11_	[M + COOH]^−^	479.1202	−0.77	−: 299.0678,255.0786,161.0356	1.6304	3.61E-07	↑
23	14.01	Betulalbuside A	C_16_H_28_O_7_	[M + Na]^+^, [M + COOH]^−^	355.1727	−1.47	+:254.0916,201.1956,153.8828	1.869	0.000001	↓
					377.1819		−:282.1093,242.1579,169.0120			
24	14.46	Rutin	C_27_H_30_O_16_	[M + H]^+^, [M − H]^−^	611.1611	−0.75	+: 493.0648, 303.0476,153.0173	1.5921	0.000009	↓
					609.1462		−:465.1028,303.0520,163.0413			
25	14.92	Quercetin-3-O-glucoside	C_21_H_20_O_12_	[M + H]^+^, [M − H]^−^	465.1026	−3.50	+:303.0566,195.0987,153.0176	1.4367	8.18E-09	↓
					463.0900		−:311.0535,179.0465,149.0205			
26	15.08	Quercetin 3-O-glucuronide	C_21_H_18_O_13_	[M + H]^+^, [M − H]^−^	479.0829,477.0686	−0.76	+: 303.0504, 257.0456,201.0549	1.6558	2.47E-08	↓
							−: 301.0359,151.0035,113.0246			
27	15.31	Kaempferol-3-O-α-L-rhamnose-(1→6)-O-β-D-glucopyranoside	C_27_H_30_O_15_	[M − H]^−^	593.1516	−0.81	−: 477.0664,301.0446,135.0181	1.6413	3.61E-06	↑
28	16.00	Kaempferol-3-O-glucoside	C_21_H_20_O_11_	[M − H]^−^	447.0930	0.72	−: 285.0388,163.0400,149.0074	1.5800	1.39E-07	↑
29	16.39	5-hydroxymethylfurfural	C_6_H_6_O_3_	[M + H]^+^	127.0379	8.91	+: 109.0320,97.0240,85.0272	1.9019	7.67E-07	↑
30	16.57	Salviaflaside methyl ester	C_25_H_28_O_13_	[M + H]^+^	537.1581	3.63	+: 331.1531, 221.1172,177.0902	1.7635	0.003971	↑
31	20.19	Quercetin	C_15_H_10_O_7_	[M + H]^+^	303.0505	−1.74	+: 257.0484,229.0452,153.0178	1.2554	0.000271	↓
32	25.07	Kaempferol	C_15_H_10_O_6_	[M + H]^+^	287.0538	3.88	+: 199.9672,153.0196,121.0280	1.3529	9.92E-12	↓
33	27.56	Ursolic acid	C_30_H_48_O_3_	[M + H]^+^	457.3670	0.59	+: 407.3275,282.0463,152.9912	1.2952	0.005619	-
34	29.35	Oleanolic acid	C_30_H_48_O_3_	[M + H]^+^	457.3678	0.70	+: 407.3300,304.0126,162.8334	1.6028	6.51E-05	↑

### 3.3 Alteration of distinguishing markers between raw CF and wine-processed CF samples

**FIGURE 4:**
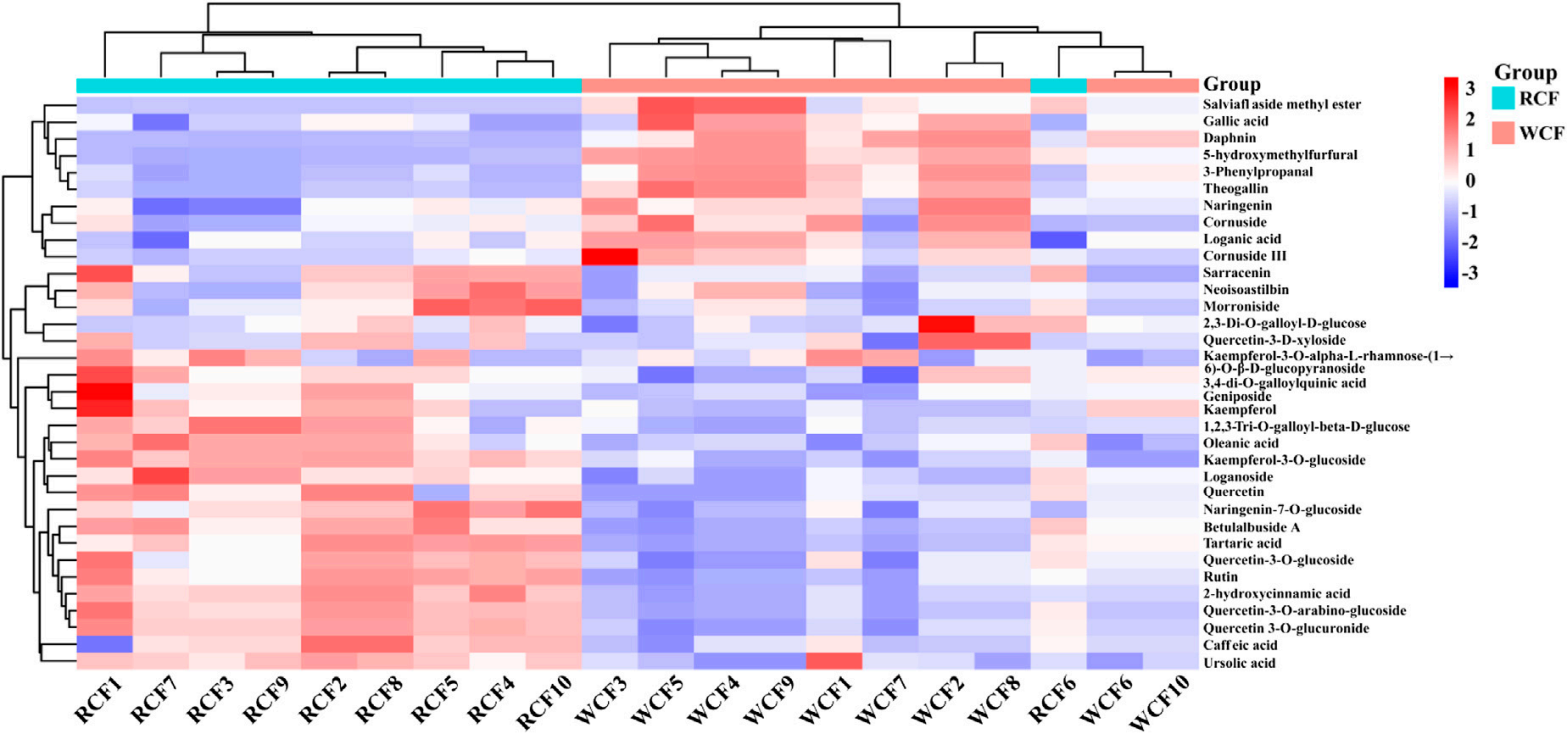
Heat map of 34 metabolites of raw CF and wine-processed CF.

**FIGURE 5:**
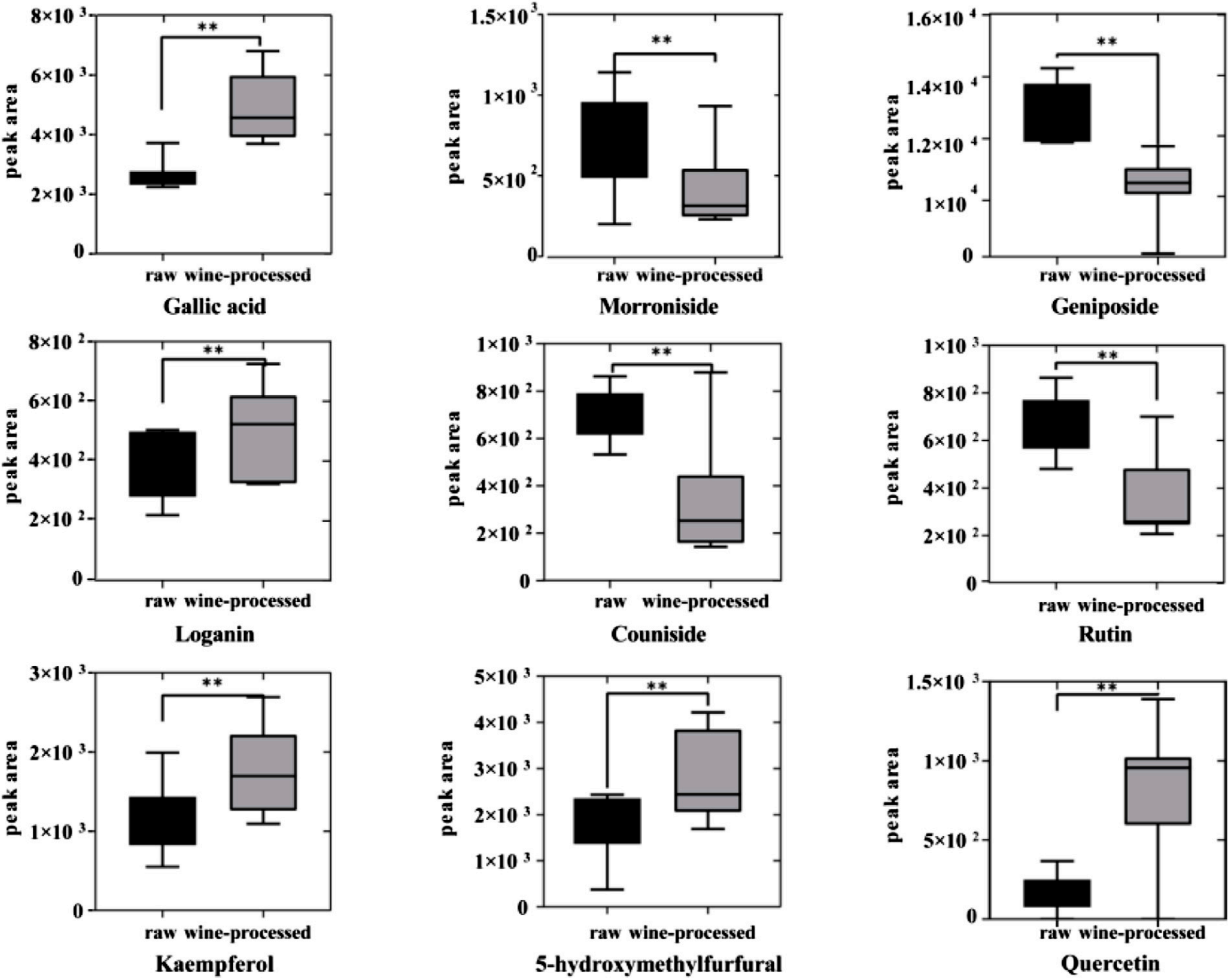
The relative content changes of 9 metabolic markers.

To assess the differences between raw CF and wine-processed CF samples, a heatmap was generated for the aforementioned potential biomarkers (Figure 4). The color variations observed in Figure 4 between the groups of raw CF and wine-processed CF indicate alterations in metabolites following wine-processing. As shown in Figure 5, four metabolites (geniposide, morroniside, rutin, and cornuside) experienced a significant decrease in wine-processed CF, while five other metabolites (loganin, gallic acid, quercetin, 5-hydroxymethylfurfural, and kaemperfol) increased (Table 2). The remaining 25 markers change plots were shown in Supplementary Figure S4. This phenomenon may be attributed to the compound’s poor stability, as they underwent hydrolysis and aglycon bond breakage under high temperatures. For example, morroniside can transform into sarracenin by losing one sugar moiety. As the wine-processing progresses, the structure of morroniside would be disrupted. Notably, the levels of gallic acid increased in wine-processed CF samples, suggesting a potential transformation of cornuside into gallic acid.

**TABLE 2:** The content of 9 markers in different bathes of Corni Fructus samples (mg/g).

Compounds	Gallic acid	Geniposide	Morroniside	Loganin	Cornuside	Quercetin	Kaempferol	Ursolic acid	Oleanolic acid
S1	0.678	0.155	5.548	3.650	0.757	0.017	0.002	0.020	0.083
S2	0.692	0.160	5.314	3.570	0.761	0.017	0.002	0.010	0.096
S3	0.731	0.134	5.491	3.693	0.733	0.013	0.002	0.013	0.022
S4	0.743	0.146	5.506	3.717	0.747	0.014	0.002	0.018	0.040
S5	0.538	0.140	5.172	3.757	0.747	0.005	0.001	0.023	0.050
S6	0.538	0.139	5.218	3.785	0.764	0.005	0.001	0.024	0.054
S7	0.664	0.165	5.121	3.497	0.727	0.012	0.002	0.042	0.150
S8	0.686	0.192	5.222	3.625	0.712	0.013	0.002	0.039	0.114
S9	0.662	0.186	4.940	3.861	0.781	0.005	0.001	0.034	0.094
S10	0.681	0.190	4.179	3.654	0.701	0.004	0.002	0.031	0.008
P1	1.557	0.070	3.441	5.143	0.618	0.013	0.008	0.005	0.004
P2	1.505	0.073	3.218	5.177	0.671	0.014	0.008	0.007	0.004
P3	1.538	0.094	3.506	5.141	0.503	0.018	0.006	0.005	0.003
P4	1.587	0.097	3.471	5.267	0.556	0.014	0.005	0.006	0.006
P5	1.156	0.078	3.631	4.140	0.541	0.014	0.006	0.007	0.006
P6	1.083	0.076	3.474	4.118	0.561	0.014	0.009	0.006	0.012
P7	1.525	0.069	3.756	4.084	0.628	0.165	0.005	0.017	0.023
P8	1.538	0.071	3.459	4.029	0.638	0.174	0.008	0.015	0.025
P9	1.503	0.070	3.640	4.090	0.632	0.014	0.005	0.016	0.029
P10	1.571	0.095	3.111	4.006	0.692	0.017	0.009	0.006	0.027

### 3.4 Network analysis of chemical composition

**FIGURE 6:**
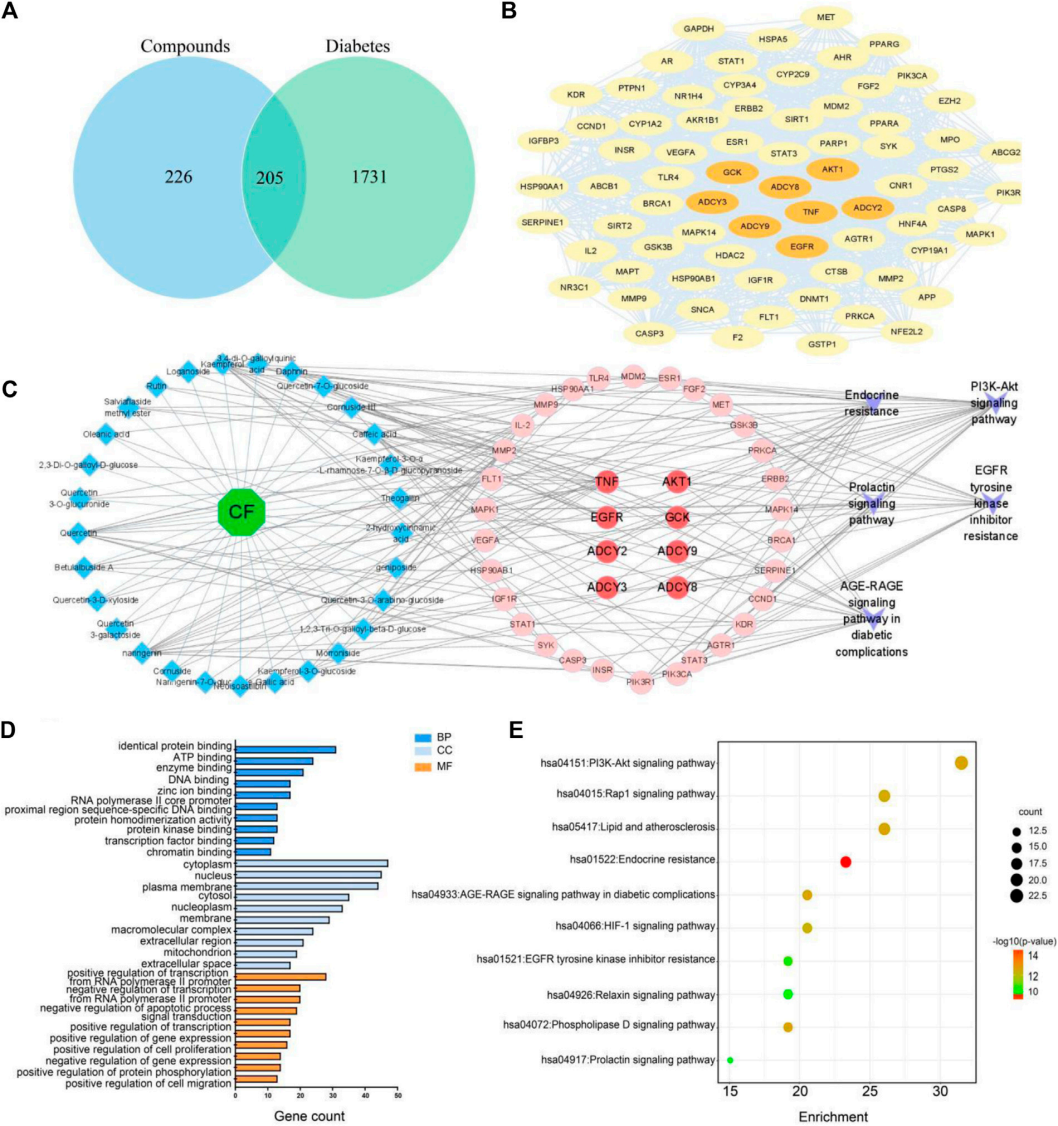
Venn diagram of potential targets **(A)**. PPI network diagram **(B)**. A comprehensive metabolites-targets-pathway interaction network **(C)**. GO enrichment analysis of potential targets **(D)**. KEGG pathway enrichment analysis **(E)**.

To further investigate the potential targets and mechanisms of hypoglycemic markers, a total of 34 markers were predicted as 431 targets from the TCMSP database and the Swiss Target Prediction. Subsequently, 1936 diabetes-related targets were collected by the DisGeNET, GeneCards, and OMIM databases. A total of 205 targets were found to be mutual between markers-related targets and diabetes-related targets (Figure 6A). Among these, 205 targets were used to construct a PPI network using the STRING website. The “Analyze Network” tool in Cytoscape 3.9.1 was used to screen the targets in PPI according to the degree values. As a result, 73 hub targets were selected, and the core PPI network consisted of 73 nodes and 1,106 edges (Figure 6B). The hub targets are presented in Supplementary Table S3.

Furthermore, the hub targets were subjected to Gene Ontology (GO) and Kyoto Encyclopedia of Genes and Genomes (KEGG) enrichment analyses using the DAVID database. The results are presented in Figure 6D, where the top 10 categories for biological process (BP), cellular component (CC), and molecular function (MF) are visually depicted. Biological process analysis revealed that CF primarily regulated glucose metabolic processes, glucose homeostasis, and cellular responses to vascular endothelial growth factor stimulus. The molecular function analysis suggested that CF mainly influenced transcription factors, enzymatic binding, and energy-related activities. In terms of cellular component, the targets regulated by CF predominantly involved in the cytoplasm, plasma membrane, and nucleus. The KEGG pathway enrichment analysis of these shared targets highlighted the significant regulation of CF on the PI3K-Akt signaling pathway, Rap1 signaling pathway, and endocrine resistance pathway (Figure 6E; Supplementary Table S4. To display the interplay between CF metabolites, targets, and pathways, a comprehensive metabolites-targets-pathway network was constructed using Cytoscape software. This network consisted of twenty-nine metabolites, eight targets, and five pathways (Figure 6C). To further screen the core components, we conducted the network topology analysis. Speculatively, eight potential active metabolites, including kaempferol, quercetin, oleanolic acid, ursolic acid, loganin, geniposide, morroniside, cornuside, were implied as key bioactive constituents, EGFR, AKT1, ADCY2, ADCY3, ADCY8, ADCY9, and GCK were possible to be the core targets which played important roles in CF against diabetes. Collectively, these results hinted that CF might exert its hypoglycemic effect through the modulation of endocrine metabolism.

### 3.5 Effect of active ingredients on HepG2 cells viability

To investigate the hypoglycemic activity of the active ingredients on HepG2 cells, we evaluated the cytotoxicity of these compounds (kaempferol, quercetin, oleanolic acid, ursolic acid, loganin, and morroniside) on HepG2 cell growth. The results showed that these tested compounds exhibited significant cytotoxicity towards HepG2 cells at concentrations ranging from 0.01 *μ*M to 10 *μ*M (Supplementary Figure S6). Subsequently, glucose consumption experiments were conducted within this concentration range.

### 3.6 Effect of active ingredients on glucose consumption in HepG2 cells

The glucose consumption effect of the active ingredients was evaluated. Compared to the control group, the positive drug group treated with metformin showed a significant increase in glucose consumption in HepG2 cells. These tested compounds also demonstrated the ability to promote glucose consumption in HepG2 cells, with a stronger effect observed at higher concentrations. Specifically, at a concentration of 10 *μ*M, these tested compounds showed the strongest promotion effect on glucose consumption (Supplementary Figure S7). These findings suggest that all these tested compounds have the potential to enhance glucose consumption in HepG2 cells.

### 3.7 Protein expression of GCK and ADCYs

**FIGURE 7:**
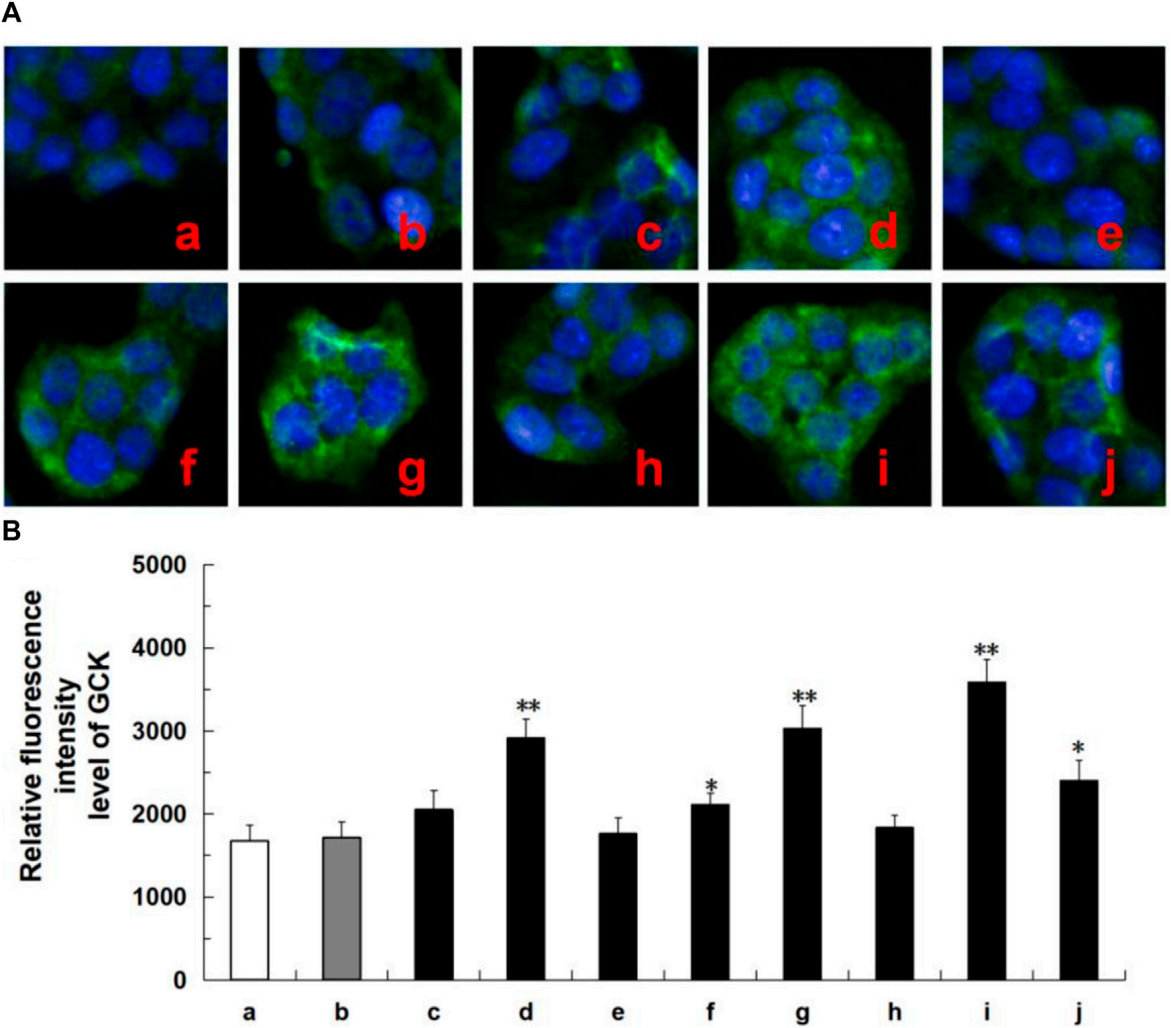
Protein expression of ADCYs (ADCY2, ADCY3, ADCY8, ADCY9) in ten tested groups by immunoluorescence staining and relative luorescence intensity. Representative confocal images **(A)** and relative luorescence intensity of GCK **(B)**. Mean ± SD, *n* = 3. **p < 0.01, *p < 0.05 vs. control group. a, control; b, dimethylsulfoxide; c, kaempferol; d, oleanolic acid; e, loganin; f, quercetin; g, ursolic acid; h, morroniside; i, metformin; j, active components of CF; DMSO, dimethylsulfoxide. Image magniication: ×200.

**FIGURE 8:**
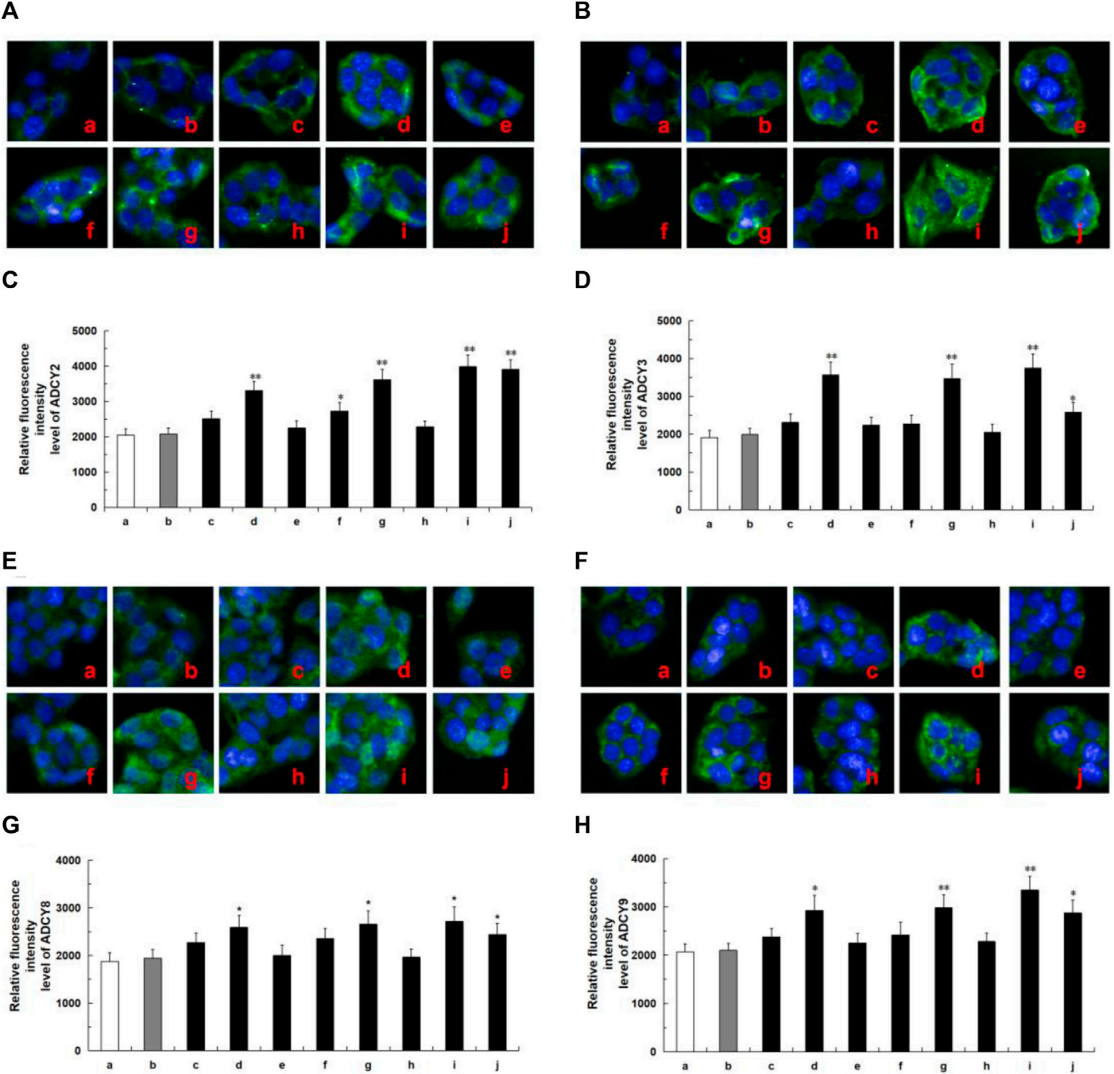
Protein expression of ADCYs (ADCY2, ADCY3, ADCY8, ADCY9) in ten tested groups by immunoluorescence staining and relative luorescence intensity. Representative confocal images **(A)** and relative luorescence intensity of ADCY2 **(C)**. Representative confocal images **(B)** and relative luorescence intensity of ADCY3 **(D)**. Representative confocal images **(E)** and relative luorescence intensity of ADCY8 **(G)**. Representative confocal images **(F)** and relative luorescence intensity of ADCY9 **(H)**. Mean ± SD, *n* = 3. **p < 0.01, *p < 0.05 vs. control group. a, control; b, dimethylsulfoxide; c, kaempferol; d, oleanolic acid; e, loganin; f, quercetin; g, ursolic acid; h, morroniside; i, metformin; j, active components of CF; DMSO, dimethylsulfoxide. Image magniication: ×200.

Network analysis revealed that these tested compounds of CF potentially exert a hypoglycemic effect by regulating the core proteins GCK and ADCYs, thereby affecting multiple pathways. According to the literature, glucokinase (GCK) and adenylate cyclases (ADCYs) proteins are considered important targets for diabetes treatment, glucokinase (GCK) serves as the initial and rate-limiting step in pancreatic and hepatic glycolysis (Li et al., 2022), while adenylate cyclases (ADCYs) are involved in the development of diabetes (Abdel-Halim et al., 2020). To elucidate the hypoglycemia molecular mechanism of the active ingredients in HepG2 cells, we investigated the effects of six compounds on the expression of proteins associated with GCK and ADCYs. As shown in Figure 7, oleanolic acid, quercetin, ursolic acid, and metformin groups exhibited higher levels of GCK protein compared to the control group. Likewise, in Figure 8, oleanolic acid, ursolic acid, and metformin treatments led to significant upregulation the proteins of ADCYs, and quercetin specifically upregulated the protein expression of ADCY2. These results showed multi-component of CF may exert hypoglycemic activity via upregulation of GCK and ADCYs.

## 4 Discussion

Currently, the hypoglycemic effect of CF’s active ingredients is influenced by various factors, such as processing time, processing methods, and extraction methods (Fan et al., 2022; Huo et al., 2017). Despite extensive research about the hypoglycemic activity of CF, there are still knowledge gaps relating to hypoglycemic metabolites, factors influencing the hypoglycemic effect, and the hypoglycemic mechanism before and after CF wine processing (Qian et al., 2022). Therefore, our study employed metabolomics combined with network analysis to discover bioactive metabolites in raw CF and wine-processed CF, and to explore the underlying hypoglycemic mechanisms.

The combination of metabolomics and systems pharmacology has proven to be a successful strategy for discovering the markers of TCM (Jiang et al., 2022). Used metabolomics and network pharmacology to analyze the quality markers of *Sophora flavescent* Alt., and successfully screened, quantified, and verified six potential markers as the most influential compounds (Chen et al., 2020). Used metabolomics and network pharmacology to examine the effects of Frankincense processing (Ning et al., 2018). In our results, a total of 34 markers were identified, with significant changes observed in iridoids during the processing process. During wine processing, the C-7 position in the iridoids chemical structure could be readily cleaved, the C-4 position could be easily substituted with hydroxyl groups, and the formation of glycosides by the disaccharide group becomes favorable under high temperatures and enzymatic action. Previous studies have reported that iridoids have the potential to enhance insulin resistance and ameliorate lipid metabolism disorders *in vivo* (Wang et al., 2020a; Gao and Feng 2022). Additionally, they exhibited blood glucose-lowering effects and effectiveness in combating diabetic complications (Kong et al., 2021). Moreover, significant changes in the composition of flavonoids were observed after wine processing of CF. Specifically, kaempferol-3-O-α-L-rhamnoside was firstly discovered to generate during wine processing, while the presence of kaempferol-3-O-α-L-rhamnose-(1→6)-O-β-D-glucopyranoside was nearly undetectable based on our findings. Furthermore, through the analysis of downstream metabolites, we compared the levels of the kaempferol glycoside in raw CF and wine-processed CF, and observed a significant increase in kaempferol content following wine production. Thus, we cautiously propose that flavonoid glycosides might underwent deglycoside during the wine-process, converting into flavonoid aglycon. Further investigation is needed to determine the potential correlation between these changes in chemical composition and the variation in hypoglycemic effects.

Network analysis showed that the significance of iridoids, flavonoids, and triterpenoids might be hypoglycemic active compounds. Flavonoids have been reported to enhance glucose uptake, inhibit aldose reductase, and stimulate insulin secretion (Kappel et al., 2013). Additionally, two triterpenoids, oleanolic acid, and ursolic acid, exhibited significant inhibitory effects on *α*-glucosidase, resulting in delayed intestinal glucose absorption and subsequent hypoglycemic effects (Ding et al., 2018). In accorded with our findings, metabolomics and network analysis implied that iridoids, flavonoids, and triterpenoids might serve as potential markers associated with hypoglycemic effects during wine processing. Furthermore, GCK and ADCYs have been reported to play an important role in regulating the endocrine metabolism (Hughes et al., 2021; Tengholm and Gylfe, 2017). GCK, a pivotal enzyme in glucose metabolism (Sternisha and Miller, 2019), plays a vital role in maintaining glucose homeostasis, and GCK mutations can contribute to various monogenic glucose disorders in humans (Osbak et al., 2009). Studies have reported that CF exhibit blood glucose-lowering effects, restore hepatic GCK activity, and enhance insulin sensitivity in peripheral tissues (Moede et al., 2020). ADCYs are membrane-bound enzymes that catalyze the conversion of adenosine triphosphate to cyclic adenosine monophosphate (Tong et al., 2016). Studies suggest that ADCYs may influence glucose metabolism through glucose-coupled insulin secretion, and are useful targets for improving insulin secretion in human (Seed Ahmed et al., 2012). Meanwhile, these results showed CF metabolites significantly upregulated GCK and ADCY_S_ expression, compared to the control group, indicating that the interaction of the metabolites in a Chinese herbal concoction during the processing impelled the transformation of chemical metabolites, the efficacy of Chinese herbal medicine does not depend on one component entirely. It is the result of the combined action of a variety of indicator metabolites.

Collectively, our findings indicate that wine processing alterated chemical composition and hypoglycemic activity, the hypoglycemic of wine processed CF were correlated with the alterations of iridoids, flavonoids, and triterpenoids. Through the integration of network analysis, cell viability evaluation, and immunofluorescence assays, we have established the correlation between targets and small molecules. Nevertheless, while we acknowledging the limitations of network analysis, including uncertainty and unreliability in predicted outcomes, experimental validation is crucial to ensure result reliability. Hence, in future experiments, we aim to assess these targets using *in vivo* studies and gain deeper insights into the underlying molecular mechanisms of action.

## 5 Conclusion

In summary, a strategy by integrating network analysis and plant metabolomics was developed, to uncover the alteration of active metabolites of CF during wine processing and the potential hypoglycemic effects. Initially, 34 markers in CF were tentatively identified using UPLC-Q-TOF-MS. Subsequently, the OPLS-DA model revealed that iridoids were the primary differential metabolites during wine processing. According to the network analysis, eight compounds, namely, kaempferol, quercetin, oleanolic acid, ursolic acid, geniposide, loganin, morroniside, and cornuside, were speculated to be potential hypoglycemic metabolites of CF. Additionally, GCK and ADCYs were inferred as hypoglycemic targets. Immunofluorescence assays verified that oleanolic acid and ursolic acid could upregulate the expression of GCK and ADCYs in the HepG2 cells model, suggesting the synergistic hypoglycemic mechanism of CF via multicomponent and multitarget interactions. This research contributes to a novel understanding of the impact of processing on the metabolites and hypoglycemic activity of Chinese herbal medicine.

## Data Availability

The original contributions presented in the study are included in the article/Supplementary Material, further inquiries can be directed to the corresponding author.
