# Supplementary material for: Changes in metabolites in raw and wine processed Corni Fructus combination metabolomics with network analysis focusing on potential hypoglycemic effects
Source: Front Pharmacol. 2023 Aug 7;14:1173747. doi: 10.3389/fphar.2023.1173747 (PMC10440738; doi:10.3389/fphar.2023.1173747)
Supplement: Supplementary file 1 [file DataSheet1.doc]

***Supplementary Material***

Changes in metabolites in raw and wine processed Corni Fructus combination metabolomics with network analysis focusing on potential hypoglycemic effects

**Siqian Zhou^a,e^**^†^**, Jian Liu ^a^**^†^**, Leihong Tan^b^**^†^**, Yikun Wang^c^, Jing Li^d^, Yajing Wang^e^, Changsong Ding^e^, Hongping Long ^a,e^***

^a^Center for Medical Research and Innovation, The First Hospital of Hunan University of Chinese Medicine, Changsha, China

^b^Department of Pharmacy, The Second Hospital of Hunan University of Chinese Medicine,Changsha, China

^c^Department of Pharmacy, The Second Xiangya Hospital, Central South University, Changsha, China

^d^Department of Pharmacy, Xiangya Hospital, Central South University, Changsha, China

^e^ Hunan University of Chinese Medicine, Changsha, China

*** Correspondence:**

Hongping Long, Email: longhongping84@163.com

^†^These authors share first authorship.

## S1 LC-MS conditions

The mobile phase consisted of a gradient elution of 0.1% formic acid in water (A) and acetonitrile (B), 5–15% B at 0–5 min; 15–25% B at 5–10 min; 25–45% B at 10–20 min; 45–65% B at 20–30 min; 65–95% B at 30–40 min. The injection volume was 2 μL.

The following operation parameters were used: ion source gas 1 and ion source gas 210 psi; curtain gas, 35 psi; ion spray voltage floating, 4000 V; temperature, 350℃; collision energy, 110 V; and collision energy spread, 30 V.

## S2 Online website

TCMSP database (https://old.tcmsp-e.com/tcmsp.php)

Swiss Target Prediction (http://www.swisstargetprediction.ch/)

DisGeNET(https://www.disgenet.org/)

GeneCards(https://www.genecards.org/)

OMIM(https://omim.org/).

STRING(https://string-db.org/)

DAVID (https://david.ncifcrf.gov/)

## S3 Identification of iridoids

Peaks **9** was established as C_11_H_14_O_5_, as they gave [M+H]+ ion at *m/z* 227.0917. The fragment ions of peak **9** at *m/z* 183.0583 indicated successive loss of a COOH (45 Da). Product ions at *m/z* 155.0320 and *m/z* 109.0285 were also found for peaks **9**. All the results suggest that peaks **9** correspond to sarracenin.The possible fragmentation pathway is shown in Figure S2A. Peak **16** produced precursor ions at *m/z* 435.1613 [M+COOH]^−^ (C_17_H_26_O_10_) , and in the negative MS/MS spectrum, fragmentation of this molecule generated product ions at *m/z* 227 (C_11_H_16_O_5_) by losing a glucoside (162Da), at *m/z* 209 (C_11_H_14_O_4_) by losing a H_2_O (18Da), and at *m/z* 181 (C_13_H_9_O_2_) by losing a CO (28Da). Peak **16** was confirmed as loganin by comparing with the mass data of reference standards. The possible fragmentation pathway is shown in Figure S2B. Peaks **20** gave [M-H]_−_ ions at *m/z* 541.1582 (C_24_H_30_O_14_). In the MS/MS spectrum, peak **20** gave dominant ions at *m/z* 379.1083 with a loss of a glucoside (162Da), at *m/z* 183.0296 with a loss of H_2_O (18Da), and *m/z* at 169.0150 with a loss of CH_2_ (14Da); After comparing with the reference standards, peak **20** was identified as cornuside ,the possible fragmentation pathway is shown in Figure S2C.

## S4 Identification of flavonoids

Peaks **14** was established as naringenin-7-O-glucoside(C_21_H_22_O_10_), at *m/z* 317.0740 with a loss of C_6_H_10_O_5_ (162Da), and *m/z* at 209.0723 with a loss of C_15_H_11_O_5_ (270Da), and at *m/z* 151 .0490(C_5_H_11_O_5_) by losing a C_16_H_22_O_5_(328Da), All the results suggest that peaks **14** correspond to naringenin-7-O-glucoside. Peak **24**, **32**, **31** was confirmed as rutin, kaempferol, quercetin by comparing with the mass data of reference standard. Peak **25** was found to have a retention time of 14.92 min, producing molecular ions of *m/z* at 463.0900 ([M-H]^−^) (C_21_H_20_O_12_). The fragment ions of peak **25** at *m/z* 311.0535 indicated successive loss of a C_6_H_10_O_5_ (162Da). Product ions at *m/z* 179.0465 and *m/z* 149.0205 were also found for peaks **25**.

## S5 Chemical transformation mechanisms in Corni Fructus

Total Ion Chromatogram (TIC) of different batches of Corni Fructus extracts is shown in Figure S1. During wine-processed, iridoids contain semi-acetal structure and alkene ether bonds, which are easily hydrolyzed and broken, and are prone to further oxidative polymerization and other reactions. In this study, it can be seen that there are 7 kinds of iridoids chemical compositions that have undergone significant changes after preparation, such as sarracenin, geniposide, morroniside, loganic acid, loganin, and cornuside. After wine processed, the C-7 position in iridoids chemical structure is easily broken, the C-4 position is easily replaced by hydroxyl groups, and it is easy to desaccharide group to form glycosides at high temperatures and the action of enzymes. The chemical composition of flavonoids is mainly the removal of one glucoside, for example, naringenin-7-O-glucoside can be transformed into naringenin by losing one sugar moiety, quercetin-3-O-glucoside can be transformed into naringenin by losing one sugar moiety. The conversion pathway is shown in Supplementary Figure S3.

**
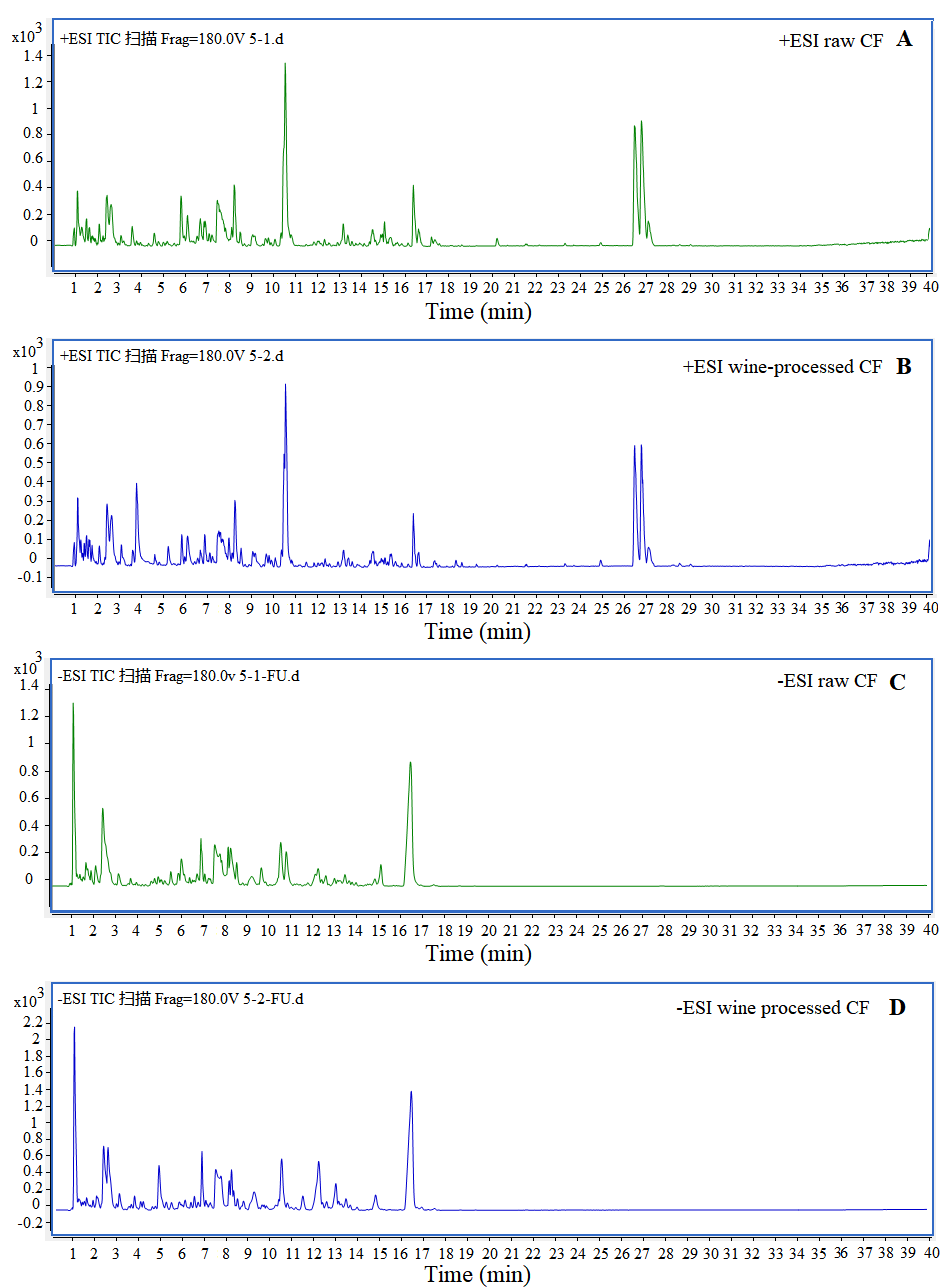
**

## Figure S1 Total Ion Chromatogram (TIC) of different batches of Corni Fructus extracts. A: ten batches of raw CF in positive ion mode; B:ten batches of wine-processed Corni Fructus in positive ion mode; C: ten batches of raw Corni Fructus in negative ion mode; D: ten batches of wine-processed Corni Fructus in negative ion mode.


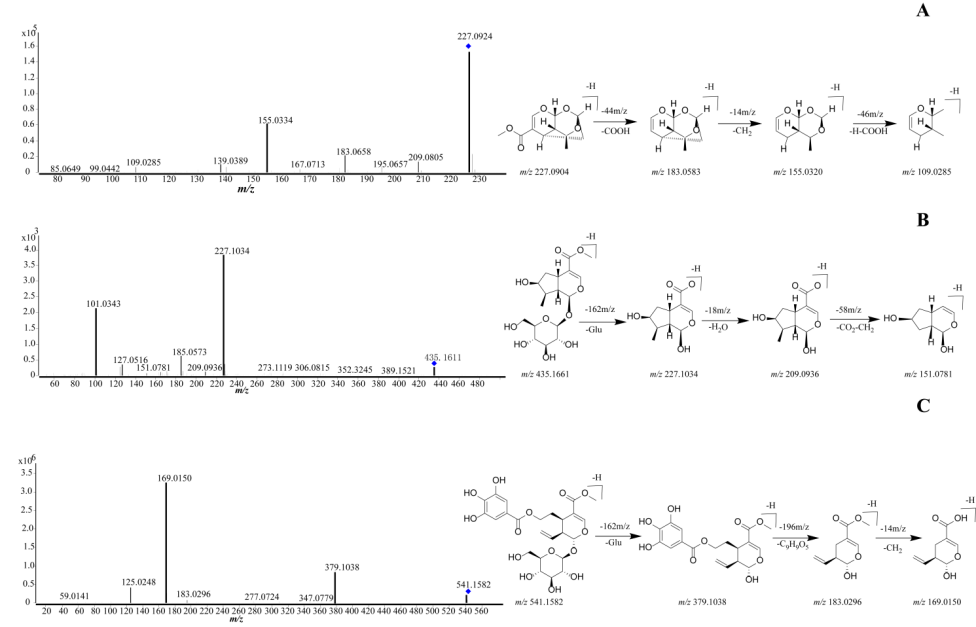


## Figure S2 The chemical structure, typical mass spectra and proposed fragmentation pathways of three types of chemical components from Corni Fructus. (A) sarracenin;(B) loganin; (C)cornuside.


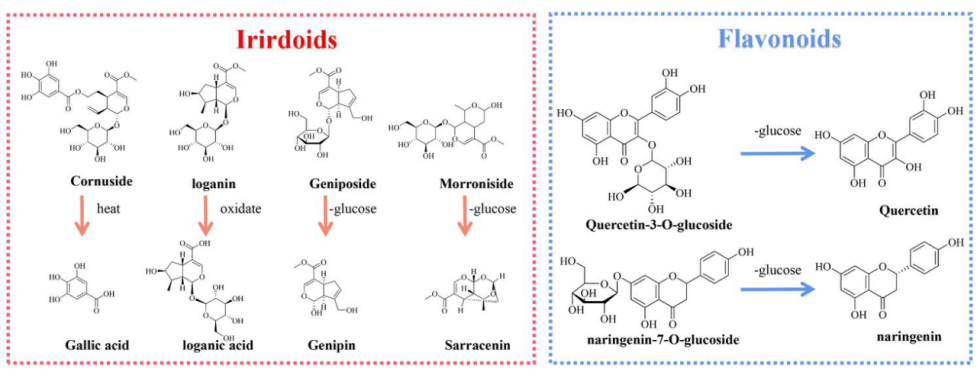


## Figure S3 Proposed processing-induced chemical transformation mechanisms of metabolites in Corni Fructus.


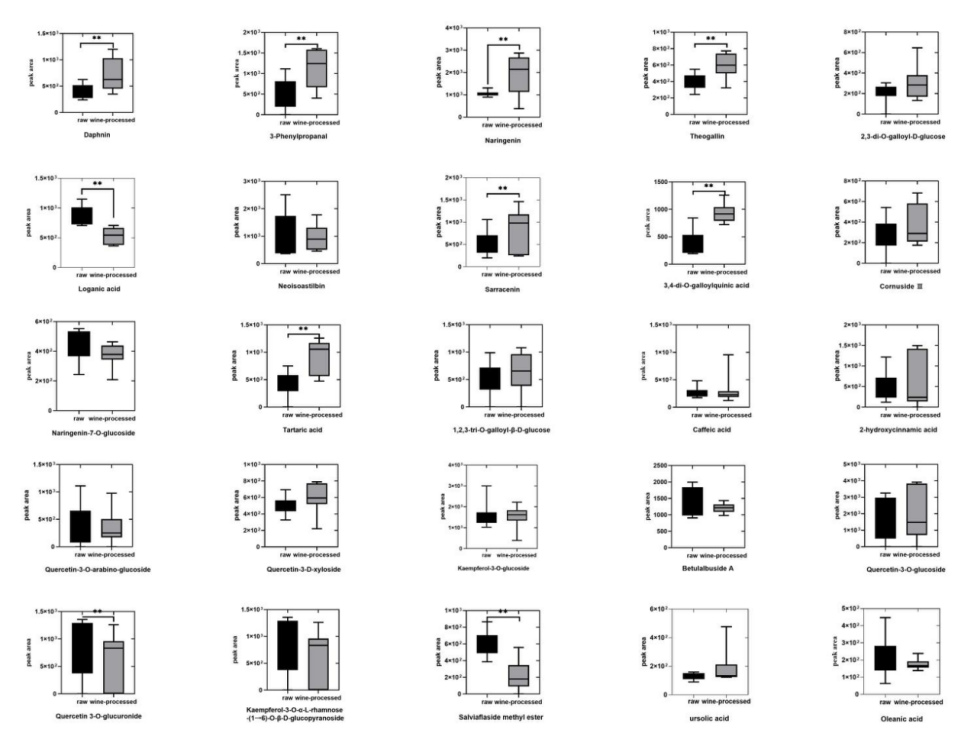


## Figure S4 The relative content changes of 25 metabolic markers in Corni Fructus.


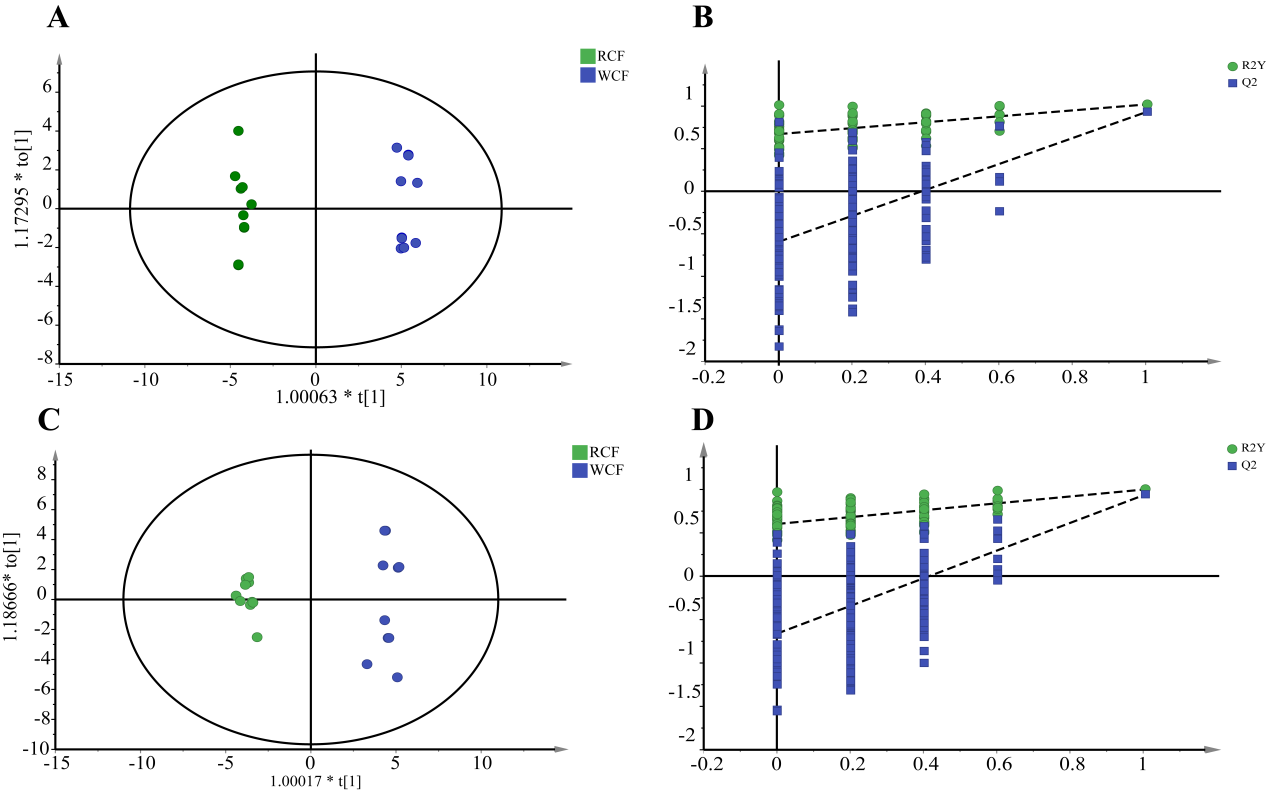


## Figure S5 (A) Positive ion mode of OPLS-DA score plot (R^2^Y=0.996, Q^2^=0.912). (B) Positive ion mode of OPLS-DA permutation test. (C) Positive ion mode of OPLS-DA score plot (R^2^Y =0.993, Q^2^ =0.935). (D) Negative ion mode of OPLS-DA permutation test.


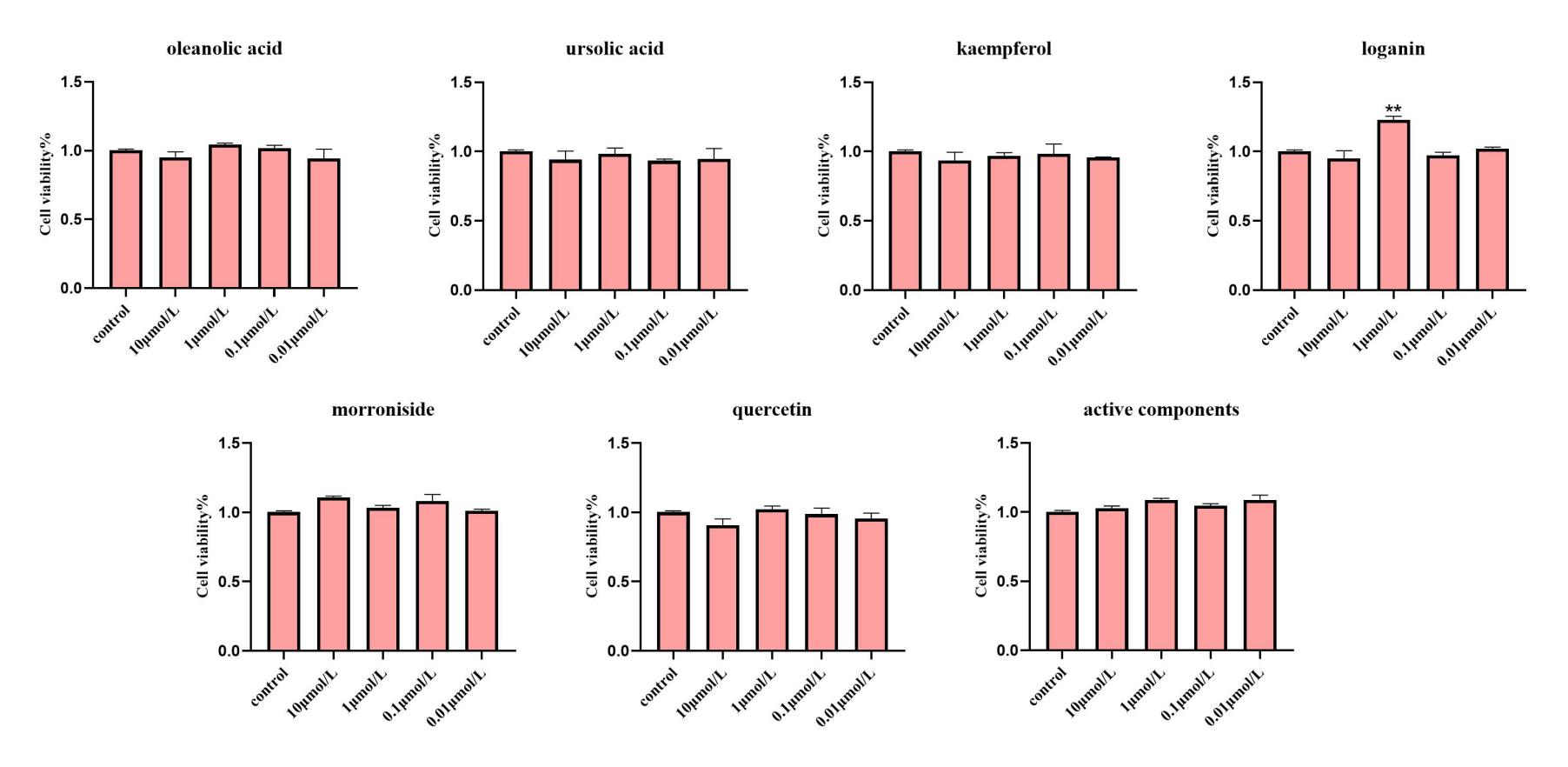


## Figure S6 Compounds had no cellular toxicity on HepG2 cells under 10 *μ*M. Data are presented as mean ± SD (n=3).


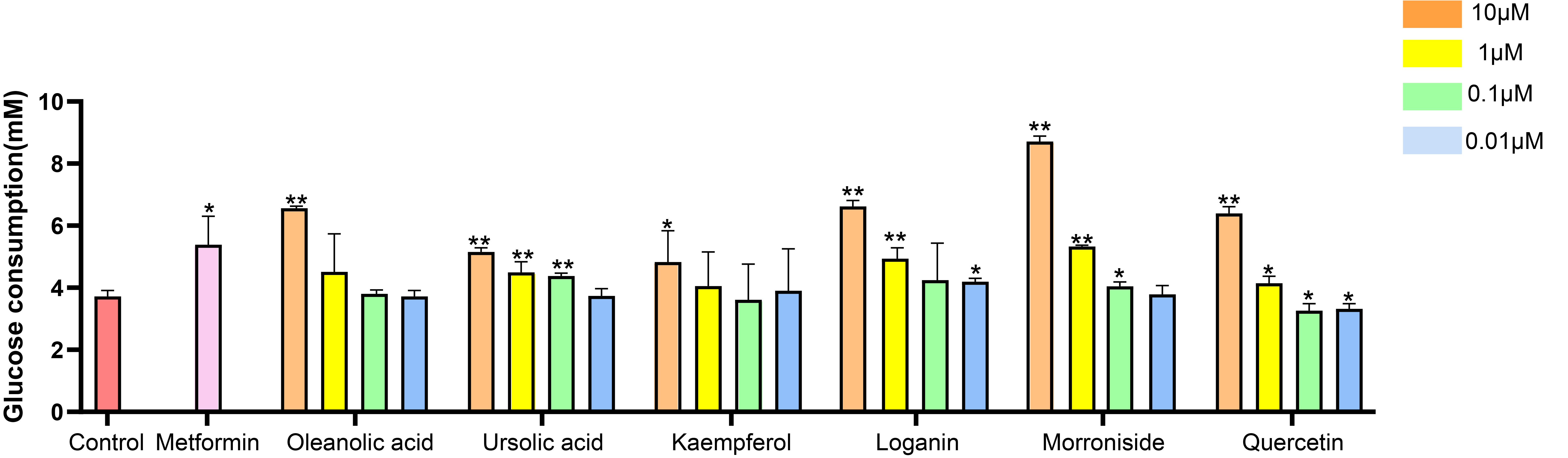


## Figure S7 Effect of compounds on glucose consumption of HepG2 cells. Data are presented as mean±SD, n= 3. **P* < 0.05, ***P* < 0.01 relative to control.

## Table S1 Medicinal material collection.

| **Species** | **Sample** | **Origins** | **Batch** | **Place of Origin** |
| --- | --- | --- | --- | --- |
| **raw Corni Fructus** | **S1** | Hunan Xinhui Pharmaceutical Co., Ltd | 20190804-1S | Xixia City, Henan Province |
|  | **S2** | Hunan Xinhui Pharmaceutical Co., Ltd | 20191211-2S | Xixia City, Henan Province |
|  | **S3** | Hunan Sanxiang Traditional Chinese Medicine Slices Co., Ltd | 20200702-1S | Chunan City, Zhejiang Province |
|  | **S4** | Hunan Sanxiang Traditional Chinese Medicine Slices Co., Ltd | 20200820-2S | Chunan City, Zhejiang Province |
|  | **S5** | Hunan Huaxia Xiangshi Pharmaceutical Co., Ltd | 20191104-1S | Hanzhong City, Shanxi Province |
|  | **S6** | Hunan Huaxia Xiangshi Pharmaceutical Co., Ltd | 20191223-2S | Hanzhong City, Shanxi Province |
|  | **S7** | Puren Traditional Chinese Medicine Slices Co., Ltd | 20200506-1S | Xixia City, Henan Province |
|  | **S8** | Puren Traditional Chinese Medicine Slices Co., Ltd | 20200506-2S | Xixia City, Henan Province |
|  | **S9** | Hunan Yirentang Traditional Chinese Medicine Slices Co., Ltd | 20200612-IS | Hanzhong City, Shanxi Province |
|  | **S10** | Hunan Yirentang Traditional Chinese Medicine Slices Co., Ltd | 20200830-2S | Hanzhong City, Shanxi Province |
| **Wine-processed Corni Fructus** | **P1** | Hunan Xinhui Pharmaceutical Co., Ltd | 20190804-1S | Xixia City, Henan Province |
|  | **P2** | Hunan Xinhui Pharmaceutical Co., Ltd | 20191211-2S | Xixia City, Henan Province |
|  | **P3** | Hunan Sanxiang Traditional Chinese Medicine Slices Co., Ltd | 20200702-1S | Chunan City, Zhejiang Province |
|  | **P4** | Hunan Sanxiang Traditional Chinese Medicine Slices Co., Ltd | 20200820-2S | Chunan City, Zhejiang Province |
|  | **P5** | Hunan Huaxia Xiangshi Pharmaceutical Co., Ltd | 20191104-1S | Hanzhong City, Shanxi Province |
|  | **P6** | Hunan Huaxia Xiangshi Pharmaceutical Co., Ltd | 20191223-2S | Hanzhong City, Shanxi Province |
|  | **P7** | Puren Traditional Chinese Medicine Slices Co., Ltd | 20200506-1S | Xixia City, Henan Province |
|  | **P8** | Puren Traditional Chinese Medicine Slices Co., Ltd | 20200506-2S | Xixia City, Henan Province |
|  | **P9** | Hunan Yirentang Traditional Chinese Medicine Slices Co., Ltd | 20200612-IS | Hanzhong City, Shanxi Province |
|  | **P10** | Hunan Yirentang Traditional Chinese Medicine Slices Co., Ltd | 20200830-2P | Hanzhong City, Shanxi Province |

## Table S2 The similarity of raw and wine-processed Corni Fructus.

| **NO.** | **Similarity** | **NO.** | **Similarity** |
| --- | --- | --- | --- |
| S1 | 0.980 | P1 | 0.949 |
| S2 | 0.980 | P2 | 0.968 |
| S3 | 0.984 | P3 | 0.913 |
| S4 | 0.980 | P4 | 0.804 |
| S5 | 0.984 | P5 | 0.936 |
| S6 | 0.749 | P6 | 0.903 |
| S7 | 0.745 | P7 | 0.886 |
| S8 | 0.980 | P8 | 0.968 |
| S9 | 0.984 | P9 | 0.804 |
| S10 | 0.984 | P10 | 0.903 |

## Table S3 73 potential target genes details of CF therapy for diabetes.

| Gene | Degree | Gene | Degree | Gene | Degree | Gene | Degree | Gene | Degree |
| --- | --- | --- | --- | --- | --- | --- | --- | --- | --- |
| AKT1 | 125 | TLR4 | 61 | APP | 44 | PRKCA | 33 | CNR1 | 27 |
| TNF | 117 | MAPK1 | 59 | BRCA1 | 43 | CTSB | 33 | SIRT2 | 27 |
| GAPDH | 114 | PPARA | 56 | CASP8 | 42 | DNMT1 | 33 | FLT1 | 27 |
| EGFR | 96 | FGF2 | 52 | PIK3R1 | 42 | HDAC2 | 33 | CYP2C9 | 26 |
| VEGFA | 95 | STAT1 | 52 | HNF4A | 40 | PTPN1 | 32 | NR1H4 | 24 |
| STAT3 | 88 | AR | 49 | SERPINE1 | 39 | NFE2L2 | 32 | CYP1A2 | 23 |
| CASP3 | 84 | IL2 | 49 | HSPA5 | 39 | ABCB1 | 31 | AKR1B1 | 21 |
| PPARG | 80 | PIK3CA | 49 | MPO | 37 | ABCG2 | 31 | AGTR1 | 20 |
| HSP90AA1 | 76 | KDR | 48 | PARP1 | 37 | SNCA | 31 | GCK | 20 |
| ESR1 | 74 | NR3C1 | 47 | CYP3A4 | 36 | F2 | 30 | ADCY3 | 14 |
| PTGS2 | 69 | MMP2 | 47 | HSP90AB1 | 36 | CYP19A1 | 30 | ADCY8 | 14 |
| CCND1 | 67 | MAPK14 | 47 | EZH2 | 36 | MAPT | 29 | ADCY2 | 11 |
| SIRT1 | 67 | GSK3B | 46 | MET | 35 | SYK | 29 | ADCY9 | 11 |
| MMP9 | 65 | MDM2 | 46 | AHR | 35 | GSTP1 | 28 |  |  |
| ERBB2 | 64 | IGF1R | 44 | INSR | 33 | IGFBP3 | 28 |  |  |

## Table S4 Combined with KEGG enrichment analysis and literature research, the top 10 pathway information was obtained.

| **NO.** | **Description** | **P-value** | **Gene count** |
| --- | --- | --- | --- |
| hsa04151 | PI3K-Akt signaling pathway | 1.21E-13 | 23 |
| hsa04015 | Rap1 signaling pathway | 1.31E-13 | 19 |
| hsa01522 | Endocrine resistance | 1.12E-16 | 17 |
| hsa04933 | AGE-RAGE signaling pathway in diabetic complications | 1.05E-13 | 15 |
| hsa04066 | HIF-1 signaling pathway | 3.58E-13 | 15 |
| hsa01521 | EGFR tyrosine kinase inhibitor resistance | 9.88E-14 | 14 |
| hsa04926 | Relaxin signaling pathway | 6.29E-11 | 14 |
| hsa04915 | Estrogen signaling pathway | 1.48E-10 | 14 |
| hsa04072 | Phospholipase D signaling pathway | 3.58E-10 | 14 |
| hsa04917 | Prolactin signaling pathway | 3.71E-10 | 11 |

## Tables S5 Core components screened by degree.

| Node_ID | Name | Degree |
| --- | --- | --- |
| MOL000422 | Kaempferol | 98 |
| MOL000098 | Quercetin | 98 |
| MOL000263 | Oleanolic acid | 75 |
| MOL000511 | Ursolic acid | 71 |
| MOL001680 | Loganin | 60 |
| MOL004102 | geniposide | 39 |
| PubChemCID:11228693 | Morroniside | 37 |
| MOL005546 | Cornuside | 29 |

## Table S6. Glucose consumption of HepG2 cells with different concentrations of compounds (n=3).

| **Compounds** | **Drug concentration( *μ*M)** | **Mean±SD** |
| --- | --- | --- |
| Control | - | 1.002±0.009 |
| oleanolic acid | 10.00 | 0.951±0.0398 |
|  | 1.00 | 1.043±0.0112 |
|  | 0.10 | 1.018±0.020 |
|  | 0.01 | 0.942±0.068 |
| Ursolic acid | 10.00 | 0.941±0.062 |
|  | 1.00 | 0.983±0.043 |
|  | 0.10 | 0.933±0.013 |
|  | 0.01 | 0.945±0.077 |
| Kaempferol | 10.00 | 0.935±0.060 |
|  | 1.00 | 0.969±0.023 |
|  | 0.10 | 0.982±0.073 |
|  | 0.01 | 0.957±0.003 |
| Loganin | 10.00 | 0.949±0.056 |
|  | 1.00 | 1.227±0.0270** |
|  | 0.10 | 0.973±0.022 |
|  | 0.01 | 1.021±0..009 |
| Morroniside | 10.00 | 1.108±0.009 |
|  | 1.00 | 1.033±0.016 |
|  | 0.10 | 1.081±0.048 |
|  | 0.01 | 1.012±0.20 |
| Quercetin | 10.00 | 0.909±0.0445 |
|  | 1.00 | 1.021±0.0248 |
|  | 0.10 | 0.987±0.0430 |
|  | 0.01 | 0.957±0.0376 |
| Active components | 10.00 | 1.027±0.0167 |
|  | 1.00 | 1.085±0.0141 |
|  | 0.10 | 1.045±0.0150 |
|  | 0.01 | 1.088±.0349 |

## Table S7. Glucose consumption of HepG2 cells with different concentrations of compounds (n=3).

| **Compounds** | **Drug concentration( *μ*M)** | **Mean±SD** |
| --- | --- | --- |
| Control | - | 3.724±0.186 |
| Metformin | 10.00 | 5.389±0.917* |
| Oleanolic acid | 10.00 | 6.559±0.074** |
|  | 1.00 | 4.512±1.229 |
|  | 0.10 | 3.807±0.118 |
|  | 0.01 | 3.724±0.186 |
| Ursolic acid | 10.00 | 5.150±0.137** |
|  | 1.00 | 4.493±0.343** |
|  | 0.10 | 4.380±0.088** |
|  | 0.01 | 3.736±0.234 |
| Kaempferol | 10.00 | 4.828±1.009* |
|  | 1.00 | 4.058±1.100 |
|  | 0.10 | 3.903±1.351 |
|  | 0.01 | 3.610±1.151 |
| Loganin | 10.00 | 6.624±0.189** |
|  | 1.00 | 4.935±0.349** |
|  | 0.10 | 4.249±1.188 |
|  | 0.01 | 4.195±0.113* |
| Morroniside | 10.00 | 8.713±0.171** |
|  | 1.00 | 5.329±0.040** |
|  | 0.10 | 4.046±0.139* |
|  | 0.01 | 3.790±0.285 |
| Quercetin | 10.00 | 6.397±0.212** |
|  | 1.00 | 4.147±0.222* |
|  | 0.10 | 3.264±0.220* |
|  | 0.01 | 3.318±0.173* |
